# Adult Onset Ornithine Transcarbamylase Deficiency: A Rare Cause of Psychosis

**DOI:** 10.1192/bjo.2024.657

**Published:** 2024-08-01

**Authors:** Hoi Ying Choi

**Affiliations:** Kwai Chung Hospital, Hong Kong, Hong Kong

## Abstract

**Aims:**

Ornithine transcarbamylase (OTC) is an enzyme of the urea cycle catalyzing the condensation of carbamyl phosphate and ornithine to form citrulline. OTC deficiency leads to elevated serum ammonia and presents as different neurological or psychiatric symptoms. OTC deficiency is an X-linked inborn error of metabolism and most cases occur in neonatal period with severe presentation. Lesser known is the late-onset form that remains latent from infancy and only presents with intriguing symptoms mimicking psychiatric disease in adulthood.

**Methods:**

Case report.

**Results:**

We describe a case of adult-onset OTC deficiency in a 40-year-old man with borderline intellectual functioning and a psychotic episode following a protein rich meal. The case was first diagnosed as undifferentiated schizophrenia, until the genetic study was carried out.

**Conclusion:**

Awareness of the adult onset ornithine transcarbamylase deficiency being a rare but possible differential diagnosis in a patient with acute psychiatric symptoms with hyperammonemia. Organic causes such as cerebral, metabolic, toxic causes of psychosis should be actively sought especially when encountering cases of acute psychosis.

